# Impact of long-term treatment of onchocerciasis with ivermectin in Kaduna State, Nigeria: first evidence of the potential for elimination in the operational area of the African Programme for Onchocerciasis Control

**DOI:** 10.1186/1756-3305-5-28

**Published:** 2012-02-07

**Authors:** Afework Hailemariam Tekle, Elizabeth Elhassan, Sunday Isiyaku, Uche V Amazigo, Simon Bush, Mounkaila Noma, Simon Cousens, Adenike Abiose, Jan H Remme

**Affiliations:** 1 African Programme for Onchocerciasis Control, WHO/APOC P. O. Box: 01 B.P. 549, Ouagadougou 01, Burkina Faso; 2 SightSavers Regional office for Africa, Dakar, Senegal; 3 Sightsavers Nigeria Country Office, 1 Golf Course Road, Kaduna, Nigeria; 4 No. 8 SOMTO ANUGWOM CLOSE. EKULU WEST, G.R.A. ENUGU, Enugu State, Nigeria; 5 Director of Advocacy and African Alliances, Sightsavers, PO Box 181909, Airport, Accra, Ghana; 6 African Programme for Onchocerciasis Control, WHO/APOC P. O. Box: 01 B.P. 549, Ouagadougou 01, Burkina Faso; 7 London School of Hygiene and Tropical Medicine, London UK; 8 Adenike Abiose, Sightcare International, P.O. Box 29771, Secretariat Main Office, Ibadan, Oyo State, Nigeria; 9 APOC consultant, 120 Rue des Campanules, 01210 Ornex, France

**Keywords:** Onchocerciasis, elimination, APOC, epidemiological evaluation, Kaduna Nigeria

## Abstract

**Background:**

Onchocerciasis can be effectively controlled as a public health problem by annual mass drug administration of ivermectin, but it was not known if ivermectin treatment in the long term would be able to achieve elimination of onchocerciasis infection and interruption of transmission in endemic areas in Africa. A recent study in Mali and Senegal has provided the first evidence of elimination after 15-17 years of treatment. Following this finding, the African Programme for Onchocerciasis Control (APOC) has started a systematic evaluation of the long-term impact of ivermectin treatment projects and the feasibility of elimination in APOC supported countries. This paper reports the first results for two onchocerciasis foci in Kaduna, Nigeria.

**Methods:**

In 2008, an epidemiological evaluation using skin snip parasitological diagnostic method was carried out in two onchocerciasis foci, in Birnin Gwari Local Government Area (LGA), and in the Kauru and Lere LGAs of Kaduna State, Nigeria. The survey was undertaken in 26 villages and examined 3,703 people above the age of one year. The result was compared with the baseline survey undertaken in 1987.

**Results:**

The communities had received 15 to 17 years of ivermectin treatment with more than 75% reported coverage. For each surveyed community, comparable baseline data were available. Before treatment, the community prevalence of *O. volvulus *microfilaria in the skin ranged from 23.1% to 84.9%, with a median prevalence of 52.0%. After 15 to 17 years of treatment, the prevalence had fallen to 0% in all communities and all 3,703 examined individuals were skin snip negative.

**Conclusions:**

The results of the surveys confirm the finding in Senegal and Mali that ivermectin treatment alone can eliminate onchocerciasis infection and probably disease transmission in endemic foci in Africa. It is the first of such evidence for the APOC operational area.

## Background

Onchocerciasis is a vector-borne parasitic disease caused by the filarial worm *Onchocerca volvulus*. The disease is endemic in Central and South America and the Yemen but 99% of the disease occurs in sub Saharan Africa, where it causes blindness and skin disease [1]. It is a disabling disease that causes significant morbidity, psychosocial problems and reduced work, especially reduced agricultural productivity in populations affected by the disease. About 37 million people in tropical Africa and 140,000 others in Latin America are infected with *O. volvulus *[1,2]. In many endemic countries including Nigeria, onchocerciasis constitutes a major public health and socio-economic problem because of its dermal and ocular manifestations. The main strategy for control in endemic countries is by mass ivermectin (Mectizan^®^) distribution. Following the availability of ivermectin and its donation free of charge by Merck and Co. to all who need it for as long as necessary [3], the control programmes in Africa adopted mass ivermectin distribution using community-directed treatment as its main control strategy [4].

Large scale distribution of ivermectin started in Kaduna State in 1991 through the community-based method in a tripartite agreement between Sightsavers, Kaduna State Ministry of Health and National Eye Centre, Kaduna to ensure delivery to all endemic communities. In 1997, a new partnership between Sightsavers, Kaduna State Government, Local Governments and endemic communities with funding from the African Programme on Onchocerciasis Control (APOC) was set up to implement Community Directed Treatment with Ivermectin distribution (CDTI).

Several studies have demonstrated the effectiveness of ivermectin as a microfilaricide, safe and good for mass dosing with major improvement in some ocular manifestations of the disease [5-7]. By reducing the microfilarial load in the infected individuals, ivermectin treatment prevents onchocercal blindness and skin disease, and reduces transmission of the parasite but cannot interrupt transmission after the first few years of treatment [8,9]. Modelling studies have predicted that elimination of infection and interruption of transmission is possible with annual ivermectin treatment, but that it may take over 25 years of treatment in hyperendemic areas [10]. However, while it has been reported that a single dose of ivermectin is not macrofilaricidal, histological evidence indicates that the release of microfilariae (mf) by adult female worms is inhibited for a period of up to one year, resulting in degeneration of intra-uterine mf and that repeated treatment may increase the mortality of adult worms [11,12]. These findings gave room to speculate that repeated doses of ivermectin over several years may have a cumulative effect on the fecundity and longevity of adult worms, and thus enhance the feasibility of elimination of onchocerciasis infection and transmission in the long term.

APOC's support to countries for implementation of CDTI in 17 African countries in 2008 resulted in annual treatment of 68.4 million people at risk [13], and elimination of onchocerciasis as a public health problem now appears within reach [14]. Despite this enormous achievement in control of onchocerciasis in Africa, there were doubts if ivermectin could also be used to eliminate infection and reduce transmission to levels at which treatment with ivermectin could be safely stopped. Many scientists had misgivings as to whether onchocerciasis elimination with ivermectin is feasible in Africa, which has more than 99% of the global cases of infection. A 2002 conference on the eradicability of onchocerciasis concluded that onchocerciasis elimination may be feasible in the Americas, where a strategy of bi-annual treatment is used and onchocerciasis is very localised, but that interruption of transmission could not be achieved in most of Africa [15]. The conference recommended that more information was needed on the impact of ivermectin intervention on transmission in different settings in Africa.

In 2009, Diawara et. al. provided the first evidence of the feasibility of onchocerciasis elimination with ivermectin in Africa [16]. In a study in three hyper-endemic foci in Mali and Senegal, they showed that after 15 to 17 years of six-monthly or annual treatments, only a few infections remained in the human population and transmission levels were below predicted thresholds for elimination. Treatment was then stopped in test areas and follow-up epidemiological and entomological evaluations after 1.5 to 2 years showed no further infections or transmission had occurred.

Following this breakthrough, APOC decided to evaluate the long-term impact of ivermectin treatment in all CDTi project areas that had at least 10 years of ivermectin treatment, in order to assess the feasibility of elimination in other parts of Africa.

Two onchocerciasis foci in Kaduna State, Nigeria, had the longest history of ivermectin treatment in the APOC operational area. These foci were therefore selected for the first evaluations undertaken by APOC to determine the impact of annual ivermectin treatment on onchocerciasis infection and transmission after 15 to 17 years of treatment.

## Methods

### Selected onchocerciasis foci

**Figure 1:**
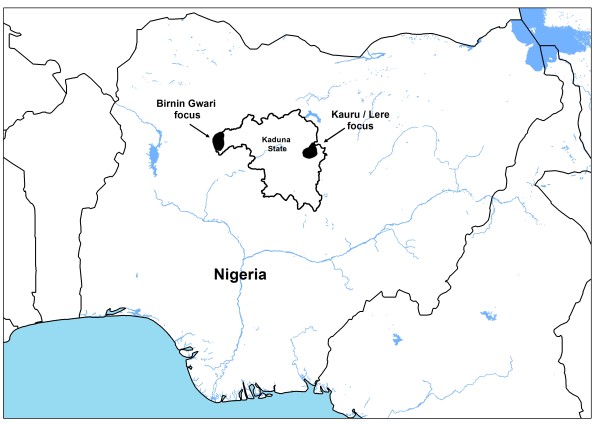
**Geographic location of the two evaluation sites in Kaduna State Nigeria**.

The evaluation was carried out in two onchocerciasis foci, one in Birnin Gwari Local Government Area (LGA), and the second in the Kauru and Lere LGAs of Kaduna State, Nigeria (Figure 1). The communities in these foci are located along the rivers of *Mairiga *and *Kwingi *in Birnin Gwari and *Galma *and *Karami *in Kauru and Lere. The two foci were selected for the following reasons: i) communities in these foci had pre-control epidemiological data; among the areas where large-scale ivermectin treatment was first introduced in Africa were these two foci in Kaduna in which treatment of a sample of the population started as part of a randomised controlled trial of ivermectin in 1988 and 1989, and where skin-snip surveys had been done in preparation for the trial [6,17]. ii) the foci included hyper-endemic villages, i.e. villages with a prevalence of microfilaridermia > 60% [15-17]; iii) the area was located along a river with known breeding sites of *Simulium damnosum s.l*., iv) the communities had had 15 - 17 years of annual treatment with ivermectin using the community-based programme since 1991, and subsequently through the community-directed treatment with ivermectin (CDTI) strategy from 1997 with more than 65% treatment coverage.

Twenty-seven communities were selected for the evaluation. These communities are mainly rural with a population range of 81 - 493 people, based on a population census undertaken during the evaluation visit in 2008 and confirmed by the leadership of the community and local administration. The people are predominantly farmers and fishermen with a few engaged in petty trading. Vegetation is mainly savannah grassland, with few areas of forest mosaic. Most of the communities, particularly in Kauru and Lere, are bordered by mountains with fast flowing rivers and streams, some of which are seasonal. The climate consists of two distinct seasons: a dry season from November to March and a wet season from April to October. During the dry season most of the rivers become dry while in areas where the rivers are perennial such as the River Galma, the blackflies are few as a result of a blackfly migration to the south of Nigeria. The rivers are reinvaded by blackfly vectors during the rainy season.

The two evaluation areas are two distinct onchocerciasis foci with the highest endemicity levels in Kaduna state. However, they are not completely isolated from neighbouring endemic areas. Along the rivers there are onchocerciasis endemic villages downstream from the evaluation areas but their endemicity levels are much lower and they are all covered by the same ivermectin treatment programme.

### Design of the evaluation

Onchocerciasis elimination is here defined as the reduction of local onchocerciasis infection and transmission to such low levels that transmission can no longer sustain itself and treatment can be safely stopped without risk of recrudescence of infection and transmission [18]. Surveillance would still be needed to detect possible reintroduction of the parasite through human or vector migration from other endemic areas where elimination has not yet been achieved.

To assess whether elimination of infection and interruption of transmission has been achieved, and ivermectin treatment can be safely stopped, APOC has adopted a phased evaluation that is based on the process used in the Mali and Senegal study [18]. During the first phase of this process, a detailed assessment is undertaken of residual onchocerciasis infection and transmission levels after at least 10 years of ivermectin treatment. The present evaluation of residual infection levels in the human population in the two onchocerciasis foci in Kaduna State represents the epidemiological component of the first phase. Where the evaluation results are satisfactory, the epidemiological evaluation will be followed by an entomological evaluation of residual onchocerciasis transmission levels during a full transmission season before any decision can be taken to stop treatment.

### Epidemiological Evaluation

In 2008, a follow-up epidemiological evaluation was conducted in 27 previously (1987) surveyed baseline villages in the two foci. In each selected village, geographic coordinates were obtained using a Global Positioning System (GPS) instrument.

Skin-snip surveys were done in all selected villages 11-12 months after the last treatment round. In each village, all subjects above the age of 1 year who agreed to participate (or whose parents agree for them to participate in the case of children) and who voluntarily presented themselves at the screening centre for the survey were asked for identification data (name, age, sex, occupation, number of years resident). The surveys used established skin-snip examination methods in which the national onchocerciasis teams had previously been trained by the WHO Onchocerciasis Control Programme (OCP). Two skin biopsies were obtained from the right and left iliac crests of all individuals who presented themselves for the survey. A 2 mm Holth corneoscleral punch (Storz instrument GMBH, Heidelberg, Germany) was used to obtain the skin biopsies. After each series of two bloodless skin-snip obtained from a subject, the scleral punch was sterilized sequentially in sodium hypochlorite solution, distilled water and then autoclaved by pressure for 15 minute. The entire process is to ensure that HIV and other blood-borne infections are not transferred. The samples were microscopically examined after incubation for 30 minutes in distilled water (and a further 24 hours in saline for negative skin-snips) for the presence and number of *O. volvulus *microfilariae [19,20]. The numbers of microfilariae were counted and the results recorded for each person examined. Information on the migration history for each person during the last 10 years before the survey was also collected.

Pre- and post-treatment skin-snip data were analyzed to determine and compare onchocerciasis infection levels using the standard indices of prevalence of microfilaria and community microfilarial load (CMFL) [21]. The CMFL was used to determine the pre-control endemicity level of each village.

### Ethical considerations

The evaluations were carried out using the WHO protocol for epidemiological surveillance and evaluation for onchocerciasis control. Ethical clearance was provided by the Kaduna State Ministry of Health after reviewing the survey protocol and instruments for data collection. Prior to the commencement of each survey; meetings were held with the local authorities and community members to sensitize them on the importance of the evaluation, mobilize them for full participation, and inform them about their right to decide to participate in the examination or not. At the point of registration for examination, verbal informed consent was obtained from all individuals, parents or a legal guardian before the commencement of the examination.

### Treatment history

Ivermectin treatment was first introduced in the two foci in 1988 as part of a randomised placebo-controlled trial of ivermectin undertaken by the Kaduna-London Onchocerciasis Collaborative Research Group to determine the impact of ivermectin treatment on visual field loss [22]. Following the successful completion of the trial, the Nigerian National Onchocerciasis Control Programme (NOCP) adopted the strategy of mass drug administration in 1991. Community-based distribution of ivermectin started in the communities between 1991 and 1993, and from 1993 onwards all communities in the two foci received ivermectin treatment. In 1997 the community-directed treatment strategy of APOC was adopted with community-selected volunteers distributing the drugs. Community treatment records show that most eligible persons have received treatment annually with therapeutic coverage above the minimum threshold of 65% in the communities assessed. Information about previous treatments was obtained from the State treatment records and verified during the field visits. Table 1 shows the treatment history of the studied villages in the three LGAs. The reported treatment coverage was 77% (range 63-86%) of the total population, or about 92% of the eligible population, which is a high treatment coverage [23].

**Table 1:** Pre-control prevalence and Ivermectin Treatment History in 27 communities in the onchocerciasis foci of Birnin Gwari and of Kauru/Lere in Kaduna State, Nigeria

Local Government Area (LGA)	Year of first Treatment	No. of evaluated communities	Pre-control prevalence of microfilaridermia (%)		No. of Treatments	Mean treatment coverage and range (%)
					
			Range	Median		
**Birnin Gwari**	1991	1	47.5	47.5	17	78.3
	1993	4	38.9-75.0	58.8	15	78.4 (76.7-79.3)
**Kauru**	1991	8	46.8-67.3	56.4	17	76.2 (74.4-80.3)
	1993	3	23.1-38.4	25.9	15	78.0 (76.5-80.4)
**Lere**	1991	5	51.3-84.6	52.7	17	77.4 (75.4-79.6)
	1993	6	30.9-55.2	37.4	15	78.1 (76.9-79.4)

Total	1991-1993	27	23.1-84.6	52.7	15-17	77.7 (74.4-80.4)

## Results

At the baseline survey (1988) a total of 6,062 subjects, out of which 2,988 (49.6%) were male and 3,038 (50.4%) female, participated in the survey. In the 2008 post-intervention survey 3,703 subjects participated of whom 1,779 (48.0%) and 1,924 (52.0%) were male and female respectively (Table 2). Out of a total census population in 2008 of 5,806 persons for the 27 villages together, 63.8% voluntarily participated in the survey. The participation rate was higher among females (66.1%) than among males (61.4%). The non-participants included children below the age of 1 year (3.5% of census population) and people who came to the examination point but then refused to participate (0.9%), but the majority (31.8%) were people who were registered as living in the village but who did not come for the examination. The non-participation rate was highest (48%) among the age group of 15 to 25 years, but dropped to around 20% from the age of 30 years onward. Between villages, the participation rate ranged from 40% to 80%.

**Table 2:** Age and sex distribution of the examined population during the baseline survey in 1987 and the evaluation survey in 2008.

Survey		Age in years					Sex		Total
		
		1-9	10-19	20-29	30-49	50+	Male	Female	N
Pre-treatment (1987)	No. examined	1,388	1,419	1,100	1,444	675	2,988	3,038	6,026
	%	23.0	23.5	18.3	24.0	11.2	49.6	50.4	100
Evaluation (2008)	No. examined	1,156	808	433	803	503	1,779	1,924	3,703
	%	31.2	21.8	11.7	21.7	13.6	48.0	52.0	100

### Onchocerciasis infection after 15-17 years of ivermectin treatment

In 1988, before treatment, 48% of the 6,026 subjects examined were positive for *O. volvulus *microfilariae in their skin-snip samples. The community prevalence of *O. volvulus *microfilaria in the skin ranged from 23.1% to 84.9%, with a median prevalence of 52.0%. The CMFL ranged from 1.2 to 8.9 mf per skin-snip, with a median of 3.9 mf overall (4.9 mf in the Birnin Gwari focus and 3.6 mf in the Kauru/Lere focus).

In 2008, after 15 to 17 years of ivermectin treatment, microfilariae could not be detected in the skin-snips from any of the 3,703 subjects who participated in the examination and the prevalence had fallen to 0% in all 27 communities (Table 3).

**Table 3:** Comparison of the results of the epidemiological surveys before treatment (1987) and after 15-17 years of treatment (2008) in the onchocerciasis foci of Birnin Gwari and of Kauru/Lere in Kaduna State, Nigeria.

Local Government Area (LGA)	Village	Total number examined		No. of people Infected		Prevalence of skin microfilariae		Community microfilarial load (mf/ss)		ONCHOSIM Predicted Prevalence
		1987	2008	1987	2008	1987	2008	1987	2008	2008
**BIRNIN GWARI** **(5)**	ANGUWAN - BAWA	220	51	154	0	70.0%	0%	4.84	0	0
	BARAU-BOKA	44	32	21	0	47.7%	0%	4.46	0	0
	ISHWAI	120	115	90	0	75.0%	0%	8.85	0	0
	KIMBI	80	118	38	0	47.5%	0%	5.22	0	0
	KURBAU	265	91	103	0	38.9%	0%	4.93	0	0

**KAURU** **(11)**	GALADIMAWA	671	235	314	0	46.8%	0%	2.87	0	0
	GARMADI	423	155	220	0	52.0%	0%	4.39	0	0
	JANKASA	124	118	83	0	66.9%	0%	3.91	0	0
	KAGUTA	88	82	58	0	65.9%	0%	8.89	0	0
	KUBAU BAUCHI	136	196	75	0	55.1%	0%	2.26	0	0
	MADAM	162	117	109	0	67.3%	0%	7.95	0	0
	SABON LAYI	134	117	76	0	56.7%	0%	3.76	0	0
	SAYAWA	108	127	25	0	23.1%	0%	1.08	0	0
	TUDUN-WADA GARMADI	276	103	106	0	38.4%	0%	2.86	0	0
	ANGUWAN - SHAWARA	247	375	64	0	25.9%	0%	1.23	0	0
	AKANSA	198	93	111	0	56.1%	0%	7.78	0	0

**LERE** **(11)**	ANGUWAN TANIMU	33	77	21	0	63.6%	0%	6.02	0	0
	ANGUWAN-BUZU	26	140	22	0	84.6%	0%	4.94	0	0
	ANGUWAN-PAH-KURAMA	96	141	35	0	36.5%	0%	3.57	0	0
	ASHEMA A	67	162	37	0	55.2%	0%	5.43	0	0
	DAN-ALHAJI	524	69	162	0	30.9%	0%	2.16	0	0
	KUDARU	524	224	269	0	51.3%	0%	4.59	0	0
	KUDURU	572	90	277	0	48.4%	0%	1.19	0	0
	UNGUWAN-PAH-HAUSAWA	164	181	53	0	32.3%	0%	1.81	0	0
	WERE I	388	67	203	0	52.3%	0%	3.73	0	0
	WERE II	281	167	148	0	52.7%	0%	1.62	0	0
	ZARANGI	55	260	21	0	38.2%	0%	2.36	0	

**Total**		**6,026**	**3,703**	**2,895**	**0**	**48.0%**	**0.0%**	**3.93**	**0**	**0**

**Figure 2:**
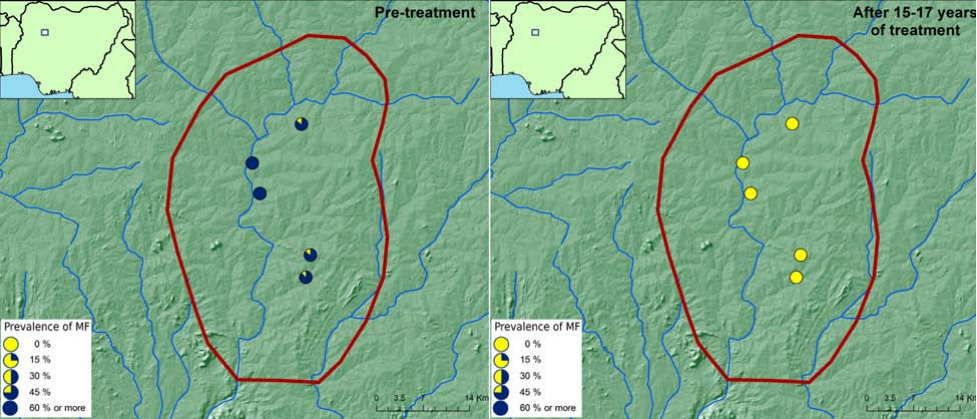
**Prevalence of onchocerciasis infection in the Birnin Gwari focus, Kaduna, Nigeria, before and after 15-17 years of ivermectin treatment**.

**Figure 3:**
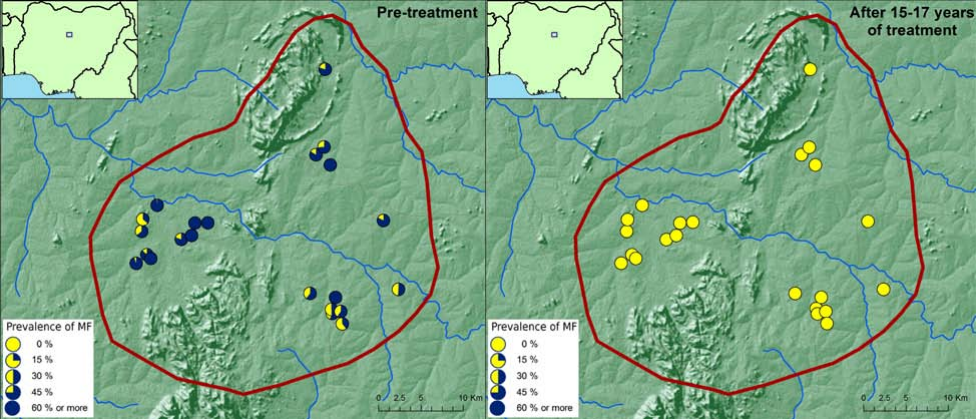
**Prevalence of onchocerciasis infection in the Kauru/Lere focus, Kaduna, Nigeria, before and after 15-17 years of ivermectin treatment**.

The results of the evaluations showed that 15-17 years of ivermectin treatment had fundamentally changed the epidemiological situation in the two foci (Figures 2 &3). While onchocerciasis was highly endemic (2 villages had a prevalence of MF > 70%) during the pre-control period in the Birnin Garbi area, after 15-17 years of treatment all villages had a microfilarial prevalence of 0%. A similar change was seen in the Kauru/Lere focus where most villages were mesoendemic, with a prevalence of between 40% and 65%, but where there were still four hyperendemic villages with a prevalence greater than 65%. Again, after treatment the epidemiology had changed fundamentally with 0% prevalence throughout the focus.

The last column in table 3 shows the prevalence that was predicted for each village using the ONCHOSIM simulation model for villages with the same pre-control endemicity level and 16 years of ivermectin treatment at 70% treatment coverage. For all villages in these two foci, the model predicted that the prevalence would have fallen to 0% after 16 years of treatment, and the observed prevalence is completely consistent with these predictions.

## Discussion

Until recently, it was doubted that onchocerciasis could be eliminated with ivermectin treatment from endemic foci in Africa, even though model predictions had indicated that elimination might be possible in the long term [10,15]. However, a recent study in three onchocerciasis foci in Mali and Senegal, where treatment had started as early as 1988/1989 with support from the former Onchocerciasis Control Programme in West Africa, showed for the first time in 2009 that elimination of onchocerciasis using long term mass drug administration of ivermectin is possible [16]. The current evaluation after 15 to 17 years of ivermectin treatment in 27 communities in two onchocerciasis foci in Kaduna State, Nigeria, has provided further evidence that elimination of onchocerciasis infection with ivermectin treatment is feasible in Africa and provides the first evidence for countries supported by APOC. Before treatment, the community prevalence of *O. volvulus *microfilaria in the skin ranged from 23.1% to 84.9% in the two foci, with a median prevalence of 52.0%. After 15 to 17 years of treatment, the prevalence had fallen to 0% in all communities and all 3,703 examined individuals were skin-snip negative. In view of previous experiences with onchocerciasis elimination in Africa, these findings are particularly significant. When the Onchocerciasis Control Programme in West Africa ceased vector control after 14 years of operations, there were still several villages with a prevalence of microfilaria between 1% and 5%, but this was not enough to maintain transmission and follow-up surveys showed that elimination had been achieved [24,25,8]. Similarly, after 15 to 17 years of ivermectin treatment in Senegal and Mali the prevalence of microfilaria had fallen to very low levels but in each focus there were still several villages with a prevalence of microfilaria between 1% and 4% [16]. However, follow-up surveys after cessation of treatment showed no evidence of renewed transmission or infection, indicating that elimination had been achieved [16,8]. Hence, the fact that in the current surveys not a single skin snip positive person was detected strongly suggest that elimination has also been achieved in the two Kaduna foci.

Nevertheless, the current evaluation involved only an epidemiological assessment of residual infection levels in the two foci and we consider the survey results only as evidence for the potential of onchocerciasis elimination.. Before cessation of ivermectin treatment can be considered, the APOC operational guidelines for onchocerciasis elimination [18] require that, in addition to the epidemiological evaluation, an entomological evaluation of residual transmission levels is done during at least one rainy season to confirm interruption of disease transmission. Such an entomological evaluation is currently ongoing and if the results confirm the absence of local *O. volvulus *transmission, the two areas in Kaduna will be the first onchocerciasis foci in the APOC operational area where ivermectin treatment will be brought to a successful conclusion.

A limitation of the epidemiological surveys is that a third of the population in the selected communities did not participate in the skin-snip examination. Though some of these had valid reasons for non-participation (age < 1 year, illness, absence from the village etc), for a large majority the reasons for non-participation were not known. This high non-participation rate could have created a bias in the survey results if those who did not participate in the survey were also more likely not to have participated in ivermectin treatment. This is another reason why an entomological evaluation of residual transmission levels is important before a final decision is taken to stop treatment in these two foci.

The treatment history is variable from village to village depending on which of the villages were included in the randomised controlled trial of ivermectin between 1988 and 1990. In the villages included in the trial, a randomly selected proportion of the population received the first treatment in 1988, and these individuals had up to 19 years of ivermectin treatment by the time of the survey. However, full community treatment with ivermectin was only introduced between 1991 and 1993, and we therefore classify these two foci as having had 15 to 17 years of ivermectin treatment.

In order to achieve elimination in an onchocerciasis endemic focus, many years of ivermectin treatment are required [10,26]. One reason for this is that the adult onchocercal parasites live for about 14 years [27]. But there are other factors that determine how many years of treatment are needed in a given focus. A critical factor is the pre-control endemicity level which reflects the initial worm load and the pre-control intensity of transmission [21,28]. As indicated in table 2 the CMFL in the villages in the two foci ranges from 1.2 to 8.9 mf per skin-snip, with a median of 3.9 mf per snip, which classifies these foci as mesoendemic [29]. The ONCHOSIM model predicts that with such low endemicity levels, onchocerciasis elimination can be achieved by 10 to 15 years of annual ivermectin treatment with good (> 75%) treatment coverage [10]. Therefore, even though we evaluated the project after 15 to 17 years of treatment with ivermectin we anticipate that the project had already achieved elimination earlier.

## Conclusion

APOC pioneered the strategy of CDTI which empowers communities to direct their own health care. This strategy appears to have achieved onchocerciasis elimination in two onchocerciasis foci in Kaduna State, Nigeria. This is the first such documented example of elimination in the APOC operational area in Africa. If the planned entomological evaluations in these two foci confirm interruption of transmission, ivermectin treatment will be stopped, making these two foci the first in any APOC country where ivermectin treatment in an onchocerciasis endemic area has been brought to a successful conclusion.

## List of abbreviations

APOC: African Programme for Onchocerciasis Control; CDTI: Community Directed Treatment with Ivermectin; CMFL: Community Microfilaria Load; GPS: Global Positioning System; LGAs: Local Government Areas; NOCP: National Onchocerciasis Control Programme; PMF: Prevalence of skin microfilariae.

## Competing interests

The authors declare that they have no competing interests.

## Authors' contributions

AHT: contributed to data cleaning, analysis, interpretation of data and write-up

EE: contributed to perform the surveys, write-up

SI: contributed to perform the surveys, write-up

UVA: contributed to design and write-up

SB: contributed to write up

MN: contributed to organizing the evaluation and write-up

AA: coordinated baseline surveys, write up

SC: provided the baseline data and contributed to write-up

JHR: contributed to design, analysis and write-up

All authors read and approved the final version of the manuscript.

## Authors' information

AHT: Medical doctor and an epidemiologist working at African Programme for Onchocerciasis Control

EE: Regional Director of SightSavers for Africa

SI: Country Director of SightSavers Nigeria

UVA: Former Director of African Programme for Onchocerciasis Control

SB: Director of Advocacy and African Alliances, Sightsavers

MN: Chief of the Unit of Epidemiology and vector Elimination at African Programme for Onchocerciasis Control

SC: Professor at London School of Hygiene and Tropical Medicine

AA: Professor and former Chair of Technical Consultative Committee of APOC

JHR: APOC consultant on onchocerciasis elimination
